# Nonessential Research in the New Normal: The Impact of COVID-19

**DOI:** 10.4269/ajtmh.20-0325

**Published:** 2020-04-24

**Authors:** Stephanie K. Yanow, Michael F. Good

**Affiliations:** 1 School of Public Health, University of Alberta, Edmonton, Canada;; 2 Department of Medical Microbiology and Immunology, University of Alberta, Edmonton, Canada;; 3 Institute for Glycomics, Griffith University, Gold Coast, Australia

With an extraordinary show of solidarity, scientists across the globe have mobilized to combat novel coronavirus disease (COVID-19). Yet, for those who could not redirect their efforts to COVID-19, research came to a sudden standstill. Researchers at many institutions were first advised to “wind down” their research activities, and then, with little warning, laboratories were closed. Simultaneously, many clinical studies on diseases other than COVID-19 were halted in their tracks.

The impacts of research shutdowns will be felt long after the pandemic. Many scientists study diseases that do not share the same obvious urgency as COVID-19 and yet take a shocking toll on human life. For example, malaria infects more than 200 million people and takes the lives of nearly half a million people, mostly young children, each year.^1^ During laboratory closures and without clinical studies, there will be no progress toward treating and preventing malaria: no progress toward new drugs, vaccines, or diagnostics. A PubMed search of articles published in 2019 alone† revealed at least 416 articles that describe preclinical studies of promising drugs, 492 articles on vaccines, and 1,023 articles on diagnostics for malaria. A “Lens” (lens.org) search revealed that in 2019, 3,350 patents were filed in the broad field of malaria. A search of clinical trials in progress‡ that were registered on clinicaltrials.gov as of April 15, 2020 listed 119 malaria drug trials, 61 malaria vaccine trials, and 66 clinical studies on malaria diagnostics. This demonstrates the level of activity in malaria research that has recently been shutdown.

There will clearly be a void in direct, measurable research outputs during and following the current hiatus imposed by the COVID-19 pandemic. But, there will be other impacts that are more qualitative yet equally profound, such as effects on our next generation of young scientists. Many students and fellows were caught off-guard by the sudden laboratory closures, ill-prepared to stop experiments on a few days’ notice. Trainees give their blood, sweat, and tears to their research projects; they now feel uncertain about their productivity, completing their degrees, and building their careers. Although supervisors are trying to keep their trainees engaged virtually, they also recognize the profound impact of lost momentum—a critical driver of research that keeps trainees motivated amid the many challenges and failures in a laboratory. Without an end in sight, it is difficult to instill confidence in trainees that the pandemic will not have major repercussions on their careers. We also cannot overlook the missed opportunities for international students to gain valuable experiences abroad. Until travel bans are lifted, there are no viable options for international students. This will be especially damaging to students from lower resource countries who perhaps stand to gain the most from international experiences. The global scientific community will pay a heavy price in untapped research potential.

Research on COVID-19 is considered essential, and rightly so, but we ask how research on other diseases was deemed “nonessential.”

Clearly, there was a calculated assessment of relative risks for people working in laboratories versus staying at home. Although the shutdown of “nonessential” laboratories aligns with public health messaging and measures imposed to contain the virus in our communities, we must recognize that this is not a simple formula. Lives are at risk because of stopping research on diseases that, in addition to COVID-19, have a major impact on public health. Most public funding systems assess research grants based on their potential for impact, and those funded are expected to contribute to healthier societies. Closing laboratories and halting clinical studies undermine this system. We recognize that we are living under exceptional circumstances and that decisions are being made considering the best interests of the public. But we argue that governments and academia must allow scientists to resume their work, considering all research essential. Personal protective equipment is already mandated to protect scientists from the hazards of laboratory work. Physical distancing in laboratories is feasible and currently implemented in laboratories working on COVID-19.

Governments are applying significant funding to COVID-19-related research. For example, as recently reported§ by the NIH, the organization has allocated ∼$1.8 billion in extramural funding for COVID-19-related research, in addition to regularly appropriated funds. However, because of the economic downturn as a result of COVID-19, we fear that governments will be tempted to divert funding from non-COVID-19 research instead of expanding the research budget to accommodate the new demands. This will further slow the discovery of new treatments, vaccines, and diagnostics for non-COVID-19 diseases. Novel coronavirus disease has become a new source of human suffering; it is not taking the place of other diseases.

Three of the leading drug candidates against COVID-19 emerged from research on malaria (hydroxychloroquine), HIV infection (lopinavir/ritonavir), and Ebola (remdesivir). Based on their in vitro activity against COVID-19 or related viruses such as SARS-CoV-1 or MERS-CoV, these drugs are currently being evaluated in clinical trials.^2^ Potential repurposing of these drugs speaks volumes about the value of research on all diseases. We must acknowledge the harm that will be caused by neglecting areas of research that are not tied to COVID-19 and ensure we balance our priorities to save lives. If we assume that health and medical research are essential to reduce morbidity and mortality, then every month that research is delayed will ultimately lead to increased suffering.

## Footnotes

† Searches were restricted to publications between January 1, 2019 and December 31, 2019. The following search terms were used: (“antimalarials”[Pharmacological Action] OR “antimalarials”[MeSH Terms] OR “antimalarials”[All Fields] OR “antimalarial”[All Fields]) AND (“in vitro techniques”[MeSH Terms] OR (“vitro”[All Fields] AND “techniques”[All Fields]) OR “in vitro techniques”[All Fields] OR “vitro”[All Fields] OR “in vitro”[All Fields]); “malaria”[MeSH Terms] OR “malaria”[All Fields]) AND (“diagnosis”[MeSH Terms] OR “diagnosis”[All Fields] OR “diagnostic”[All Fields]); (“malaria”[MeSH Terms] OR “malaria”[All Fields]) AND (“vaccines”[MeSH Terms] OR “vaccines”[All Fields] OR “vaccine”[All Fields]).

‡ Studies were listed as “not yet recruiting,” “recruiting,” “enrolling by invitation,” and “active, not recruiting.”

§ https://nexus.od.nih.gov/all/2020/04/13/covid-19-funding-and-funding-opportunities/. Accessed April 19, 2020.
